# Late gadolinium enhancement on cardiac magnetic resonance predicts coronary vasomotor abnormality and myocardial lactate production in patients with chronic heart failure

**DOI:** 10.1007/s00380-016-0816-z

**Published:** 2016-02-18

**Authors:** Tomoaki Uemura, Megumi Yamamuro, Koichi Kaikita, Seiji Takashio, Daisuke Utsunomiya, Kyoko Hirakawa, Mina Nakayama, Kenji Sakamoto, Eiichiro Yamamoto, Kenichi Tsujita, Sunao Kojima, Seiji Hokimoto, Yasuyuki Yamashita, Hisao Ogawa

**Affiliations:** 1 Departments of Cardiovascular Medicine, Graduate School of Medical Sciences, Kumamoto University, 1-1-1 Honjo, Kumamoto, 860-8556 Japan; 2 Diagnostic Radiology, Graduate School of Medical Sciences, Kumamoto University, Kumamoto, Japan; 3 Department of Cardiovascular Medicine, National Cerebral and Cardiovascular Center, Osaka, Japan

**Keywords:** Heart failure, Fibrosis, Magnetic resonance imaging, Microvascular dysfunction

## Abstract

Myocardial fibrosis and microvascular dysfunction are key determinants of outcome in heart failure (HF); we examined their relationship in patients with HF. Our study included 61 consecutive patients with HF but without coronary stenosis. All underwent gadolinium-enhanced cardiac magnetic resonance to evaluate late gadolinium enhancement (LGE) and an acetylcholine (ACh) provocation test to evaluate microvascular dysfunction. During the ACh provocation test, we sampled blood simultaneously from the coronary sinus and aortic root to compare lactate concentrations. We quantified coronary blood flow volume using an intracoronary Doppler-tipped guidewire. We detected LGE in 34 patients (LGE-positive); 27 were LGE-negative. Coronary blood flow volume increased significantly after the ACh provocation test only in LGE-negative patients (before vs. after ACh, 47.5 ± 36.8 vs. 69.2 ± 48.0 ml/min, respectively; *p* = 0.004). The myocardial lactate extraction ratio (LER) significantly decreased after the ACh test in both groups (LGE-negative, *p* = 0.001; LGE-positive, *p* < 0.001), significantly more so in the LGE-positive group (*p* = 0.017). Multivariate logistic regression analysis showed that a post-ACh LER < 0 (indicating myocardial lactate production) was a significant predictor of LGE-positivity (odds ratio 4.54; 95 % confidence interval 1.38–14.93; *p* = 0.013). In the LGE-positive group, an LGE volume greater than the median significantly predicted a post-ACh LER of <0 (*p* = 0.042; odds ratio 6.02; 95 % confidence interval 1.07–33.86). ACh-provoked coronary vasomotor abnormality is closely correlated with myocardial fibrosis in patients with HF but without organic coronary stenosis. Coronary vasomotor abnormalities in fibrotic myocardium may worsen HF.

## Introduction

Myocardial fibrosis is a key component of heart failure (HF). There is a substantial body of evidence published over the past decade that there is a strong relationship between myocardial fibrosis and worsening of HF [1–5]. The presence of late gadolinium enhancement (LGE) in cardiac magnetic resonance (CMR) as an indicator of myocardial fibrosis is well established [6–8]. The prognostic value of LGE in nonischemic-HF has been described in several studies [9–14].

The arterioles are the true intra-myocardial regulatory component of the coronary circulation, and these vessels represent the largest proportion (approximately 55 %) of the total coronary vascular resistance [15]. Increasing of lactate in the coronary circulation is a definitive sign of myocardial ischemia, and metabolism of lactate in the coronary circulation is possible to compare plasma lactate concentration in the aortic root (Ao) and coronary sinus (CS) to assess the occurrence of myocardial ischemia during coronary angiography [16, 17].

The intracoronary acetylcholine (ACh) provocation test is a sensitive, safe test for the assessment of coronary vasomotor function in the catheterization laboratory. Administration of ACh causes vasodilation under normal conditions; however, in the presence of endothelial dysfunction it leads to vasoconstriction due to decreasing of nitric oxide synthesis [18–21].

Coronary microvascular dysfunction affects the development of nonobstructive coronary disease. Microvascular dysfunction is also a significant and independent determinant of long-term cardiac events in patients with HF [22–31]. However, the correlation between coronary microvascular dysfunction and myocardial fibrosis is not fully understood. The aim of this study was to investigate the relationship between coronary microvascular circulation and LGE on CMR in patients with HF but without coronary stenosis.

## Materials and methods

### Patient characteristics

This study included 61 consecutive patients with suspected non-ischemic HF who underwent both cardiac catheterization and contrast-enhanced CMR between January 2007 and December 2013 at Kumamoto University Hospital.

Eligible patients were ≥20 years of age and met the criteria for the diagnosis of HF set by the American College of Cardiology/American Heart Association Stage B or C classification. The patients were clinically stable and under optimal medical therapy for HF, including stable doses of an angiotensin-converting enzyme (ACE) inhibitor or angiotensin II receptor blocker (ARB) and a β-blocker, if not contraindicated. Twenty-one of the 61 patients (37.7 %) were, however, unable to tolerate ACE inhibitor, ARB or beta blocker therapy because of hypotension even at low doses: seven could not tolerate an ACE inhibitor (or ARB) and beta blocker combination therapy, and seven could not tolerate either an ACE inhibitor, ARB or a beta blocker alone. Thirty-nine of 61 patients (63.9 %) had dilated cardiomyopathy (DCM) and eleven of the 61 patients (18.0 %) had hypertrophic cardiomyopathy (HCM) (Table 1).

**Table 1:** Etiology of HF

	*n* = 61
Dilated CM	39 (63.9)
Hypertrophic CM	11 (18.0)
Hypertensive heart disease^a^	5 (8.1)
Diabetic CM^b^	2 (3.2)
Others	4 (6.5)

Values are presented as *n* (%)

Other etiologies included cardiac amyloidosis (*n* = 2), Becker muscular dystrophy (*n* = 1) and thyrotoxicosis (*n* = 1)

*CM* cardiomyopathy

^a^Defined as a previous history of long-standing hypertension and presence of left ventricular hypertrophy (left ventricular mass index >101 g/m^2^ in women and >117 g/m^2^ in men) with ACC/AHA Stage B or C HF, and in the absence of a cause other than hypertension

^b^Defined as a previous history of uncontrolled diabetes (HbA1c > 6.5 %) and left ventricular dysfunction in the absence of a cause other than diabetes

The following exclusion criteria were applied during patient selection: decompensated HF, acute coronary syndrome, hypertrophic obstructive cardiomyopathy (HOCM), significant coronary stenosis (>50 %), renal insufficiency with an estimated glomerular filtration rate of <30 ml/min per 1.73 m^2^, myocarditis, muscular dystrophy, severe cardiac valvular disease, an implanted cardiac resynchronization device, and chronic inflammatory disease.

Written informed consent was obtained from each patient before cardiac catheterization. This study was approved by the Human Ethics Committee of our institution.

### Echocardiograms

Left ventricular ejection fraction (LVEF), left atrial diameter, interventricular septal wall thickness (IVSd) and posterior wall thickness (PWd) were measured by two sonographers according to the recommendations for chamber quantification of the American Society of Echocardiology [32].

*E*/*e*′ is the ratio between early mitral inflow velocity (E) and mitral annular early diastolic velocity (e′); we measured e′ at the septal mitral annulus. Left ventricular end-diastolic volume (LVEDV) and left ventricular end-systolic volume (LVESV) were calculated by either of the two methods described above; LVEF was calculated as follows:

$${\text{LVEF}} = \left( {{\text{LVEDV}} - {\text{LVESV}}} \right) \, / {\text{ LVEDV}}$$

### Protocol for CMR

CMR was performed on a 3.0 T magnetic resonance instrument (Achieva 3.0T X-series or Achieva 3.0T X-series TX; Philips Medical Systems, Best, The Netherlands). Patients were scanned in the supine position using a dedicated 6- or 32-channel cardiac torso coil, between January 2007 and December 2013.

Double-angulated scout images were obtained to plan the cardiac axis views. In all patients, images were acquired using T1- and T2-weighted black-blood, electrocardiogram-gated cine imaging by a segmented steady-state free-precession sequence in the three long cardiac axes with LGE imaging. Approximately 10 min after the injection of 0.1 mmol/kg of a gadolinium-based contrast agent (Magnevist; Bayer Healthcare, Leverkusen, Germany), we acquired two-dimensional inversion-recovery sequences that included the left ventricle from the base to the apex. The imaging parameters were: time to repeat/time to echo = 4.5/2.2 ms; time of inversion = 250–350 ms; acquisition pixel size = 0.95 × 1.13 mm; and slice thickness = 8.0 mm. The acquisition time per slice was approximately 14 s.

### Image analysis

Hyperenhanced myocardial voxels resembling LGE were defined as signal intensity higher than 6 standard deviations above the mean intensity of the region of interest, which was placed over an area of the myocardium evaluated as normal [7].

A board-certified cardiovascular radiologist blinded to the results of the cardiac catheterization study independently determined the dichotomous presence of LGE in the left ventricle. The patients were classified into LGE-positive or LGE-negative groups.

Imaging data were analyzed using commercially available post-processing software (AZE Virtual Place; AZE, Tokyo, Japan). The endocardial and epicardial borders of the myocardium were manually traced on each short-axis slice. The left ventricular volume was calculated by subtracting the endocardial volume from the epicardial volume at end-diastole. Calculations were performed by two cardiologists blinded to the clinical data. We also calculated the  %LGE as the proportion of the LGE volume in the total left ventricular volume.

### Cardiac catheterization

All vasoactive drugs, including calcium antagonists, isosorbide dinitrate, isosorbide mononitrate and nicorandil were discontinued ≥72 h before the ACh test. We performed all examinations the morning after an overnight fast. A 5 Fr or 6 Fr arterial cannula was placed in the radial or femoral artery, and a 6 Fr venous sheath was placed in the right femoral vein [27, 28, 33]. A CS catheter (Goodtec, Gifu, Japan) was advanced to the CS from the venous sheath, and its position was confirmed by contrast-enhanced fluoroscopy. We performed cardiac catheterization and an intracoronary ACh provocation test (100 µg/min). The lumen diameter of the left anterior descending coronary artery (LAD) at end-diastole was measured with a computer-assisted coronary angiographic analysis system (CAAS 5.6; Pie Medical Imaging B.V., Maastricht, The Netherlands) by calibrating the measurement with a Judkins catheter as previously reported [34]. The LAD was divided into proximal, middle, and distal segments of equal length, and each luminal diameter was measured at the center of each segment. The coronary artery diameter was measured by two blinded investigators at two time points: at baseline and 1 min after ACh provocation [18].

Blood was sampled from the CS and the Ao simultaneously to measure the plasma lactate concentration [27, 28]. The lactate extraction ratio (LER) was calculated by multiplying the coronary arteriovenous difference in the lactate concentration by 100 and dividing it by the arterial lactate concentration as follows [17]:

LER (%) = 100 × (arterial lactate concentration [mg/dl] − coronary venous lactate concentration [mg/dl])/arterial lactate concentration (mg/dl).

We defined an LER < 0 % as indicative of myocardial lactate production.

Additionally, we continuously monitored and evaluated the change of coronary blood supply by measuring the quantitative coronary blood flow volume (CBFV) using an intracoronary Doppler-tipped guidewire (FloWire; Volcano, Rancho Cordova, CA, USA) [27]. The quantitative CBFV was calculated as follows [35].

$${\text{CBFV}} = \pi \times \left( {{\text{average peak velocity}}/ 2} \right) \times \left( {{\text{vessel diameter}}/ 2} \right)^{ 2}$$

We compared the LER and CBFV before and after the ACh provocation test. We then diagnosed microvascular spasm according to the presence of myocardial lactate production and a decrease in the CBFV without epicardial vasospasm during the ACh provocation test.

After the ACh test, adenosine triphosphate was administered into a central vein at 150 µg/kg/min until maximal hyperemia was reached as determined by the coronary flow reserve (CFR). The CFR was calculated as follows [27]:

CFR = average peak velocity at maximal hyperemia/average peak velocity at rest.

### Assessment by ACh provocation test

All patients were diagnosed by cardiac catheterization as previously reported [22, 27]. Epicardial coronary artery spasm was angiographically defined as severe vasospasm indicated by >90 % lumen narrowing in any of the major coronary arteries in response to the ACh test. This definition is based on the Japanese Circulation Society’s guideline for vasospastic angina (epicardial coronary artery spasm) [18].

Patients without epicardial coronary artery spasm who were positive for lactate production were diagnosed with microvascular spasm based on a decrease in the CBFV without epicardial vasospasm, but with the occurrence of chest symptoms and ischemic changes on an electrocardiogram.

Patients who were positive for lactate production but had no decrease in the CBFV were diagnosed with microvascular dysfunction. Patients who were negative for lactate production were further assessed using the CFR. Patients with an abnormal CFR were diagnosed with microvascular coronary dysfunction and analyzed along with the patients in the microvascular dysfunction group [27].

Patients without abnormal results were considered not to have ischemic heart disease (IHD).

### Statistical analysis

Normally distributed data are presented as mean ± standard deviation or median (interquartile range). Differences between groups were examined by Student’s *t* test or the Mann–Whitney *U* test for unpaired data. Categorical values are presented as number (percentage) and were compared by the Chi-squared test or Fisher’s exact test, as appropriate. Variables with a skewed distribution were transformed logarithmically before Pearson’s correlation to fulfill the conditions required for the type of analysis performed.

Univariate logistic regression analysis was performed to identify significant parameters related to LGE-positivity. Multivariate logistic regression analysis was then performed using the forced inclusion model. The Hosmer–Lemeshow statistic was applied to assess model calibration.

A two-tailed *p* value of <0.05 was considered statistically significant. All statistical analyses were performed with SPSS, version 20 (IBM Corp., Armonk, NY).

## Results

**Fig. 1:**
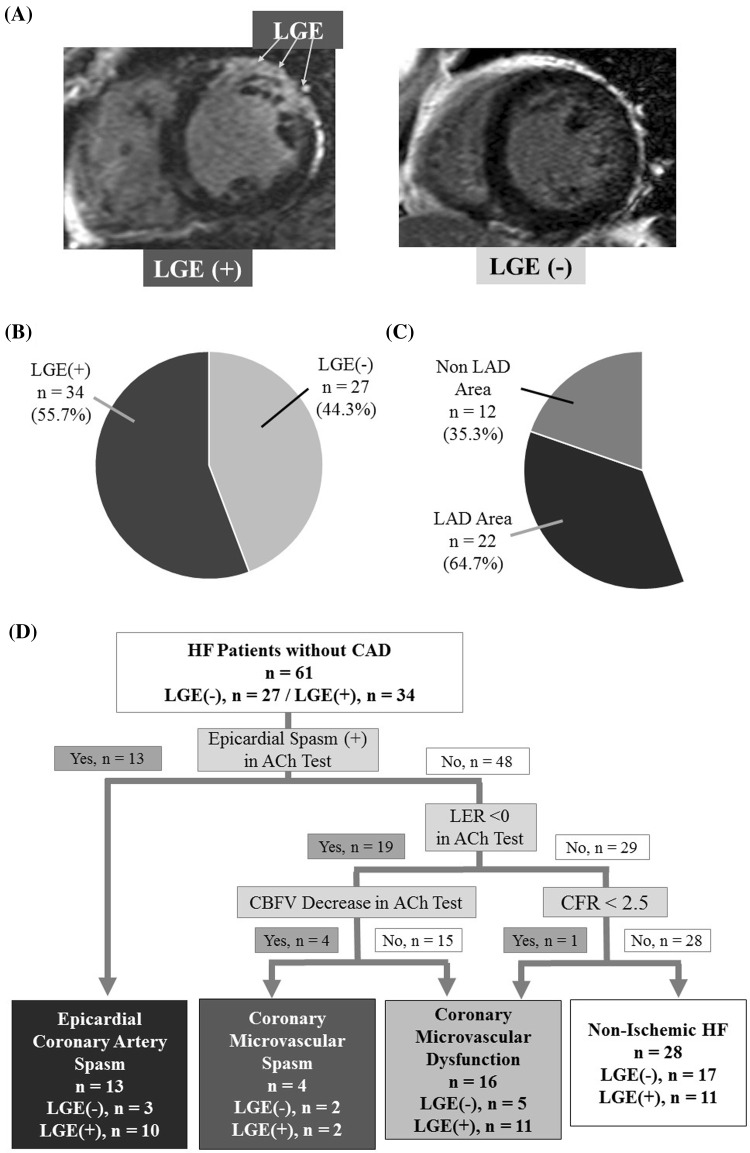
Cardiac magnetic resonance results and diagnostic flow chart with cardiac catheterization, **a** A representative image of LGE on CMR. **b** In total, 34 of 61 patients who underwent CMR exhibited LGE (LGE-positive), and the other 27 exhibited no LGE (LGE-negative). **c** 22 of the 34 LGE-positive patients exhibited LGE in the LAD coronary artery area, the remaining 12 patients exhibited LGE in other areas. *CMR* cardiac magnetic resonance imaging, *LAD* left anterior descending coronary artery, *LGE* late gadolinium enhancement. **d** Diagnostic flow chart for cardiac catheterization. All patients were initially predicted to have nonischemic-HF, but 54.0 % of patients showed nonobstructive-CAD (epicardial coronary artery spasm, coronary microvascular spasm and/or coronary microvascular dysfunction). *CAD* coronary artery disease

LGE was observed in 34 patients (LGE-positive; 55.7 %) (Fig. 1a, b). The median of  %LGE value in the LGE-positive group was 11.0 % (interquartile range 4.0–18.2 %).

We compared the baseline characteristics of the LGE-positive (*n* = 34) and LGE-negative (*n* = 27) groups. The hemoglobin and serum potassium concentrations were significantly higher in the LGE-positive group (Table 2). Next, we compared the echocardiographic findings between the two groups: E/e′ was significantly higher in the LGE-positive group (Table 3).

**Table 2:** Patient characteristics

Variable	LGE(−), *n* = 27	LGE(+), *n* = 34	*p* value
Age in years	58.6 ± 14.3	59.1 ± 16.5	0.90
Sex, male	18 (66.7)	25 (73.5)	0.56
Body mass index in kg/m^2^	23.8 ± 4.5	24.4 ± 3.9	0.61
Hypertension	17 (62.9)	20 (58.8)	0.83
Diabetes	9 (33.3)	6 (17.6)	0.17
Atrial fibrillation	11 (40.7)	7 (20.6)	0.089
Smoking history	17 (62.9)	20 (58.8)	0.89
Medications			
ACE-I/ARB	21 (77.8)	26 (76.4)	0.90
β-blocker	18 (66.7)	29 (85.3)	0.10
Loop diuretic	9 (33.3)	11 (32.3)	0.93
MR antagonist	7 (25.9)	14 (41.1)	0.21
Calcium antagonist	5 (18.5)	10 (29.4)	0.32
Laboratory data			
White blood cells (per µl)	5644 ± 2950	6111 ± 1155	0.09
Hemoglobin (in g/dl)	13.3 ± 2.1	14.8 ± 1.9	0.005
Serum albumin (in mg/dl)	3.94 ± 0.55	4.14 ± 0.42	0.11
Serum sodium (in mEq/L)	139.6 ± 2.6	139.8 ± 1.8	0.74
Serum potassium (in mEq/L)	4.24 ± 0.41	4.47 ± 0.41	0.03
Serum creatinine (in mg/dl)	0.78 ± 0.16	0.89 ± 0.23	0.052
ln(hs-TnT)	0.90 (0.25–1.56)	1.36 (0.93–1.80)	0.12
ln(BNP)	5.45 (4.17–6.72)	5.40 (4.84–5.96)	0.40

Values are presented as mean ± standard deviation, median (interquartile range) or *n* (%)

No significant differences were present in the baseline characteristics or medications between the two groups. With respect to the laboratory data, the hemoglobin and serum potassium concentrations were higher in the LGE-positive group than the LGE-negative group

*ACE-I* angiotensin converting enzyme inhibitor, *ARB* angiotensin II receptor blocker, *BNP* brain natriuretic peptide, *hs-TnT* high-sensitivity serum troponin T, *LGE* late gadolinium enhancement, *MR* mineralocorticoid receptor

**Table 3:** Echocardiography results

Parameter	LGE(−), *n* = 27	LGE(+), *n* = 34	*p* value
LVDd in mm	53.7 ± 6.5	51.9 ± 9.9	0.440
LVEF in %	43.6 ± 13.1	44.8 ± 12.9	0.716
IVSd in mm	10.8 ± 2.8	11.9 ± 3.3	0.158
PWd in mm	10.3 ± 2.9	11.1 ± 2.4	0.246
E/A	1.04 ± 0.54	1.23 ± 0.60	0.235
DcT in ms	182.2 ± 42.7	205.3 ± 86.3	0.208
E/e′	11.2 ± 3.7	*13.4* *±* *4.0*	*0.035*

Values are presented as mean ± standard deviation

*E*/*e′* was significantly higher in the LGE-positive group than the LGE-negative group

*DcT* deceleration time of mitral inflow, *E/A* ratio of early transmitral velocity (E) to late transmitral velocity (A), *E/e′* ratio of early transmitral velocity (E) to tissue Doppler early diastolic velocity (e′), *IVSd* interventricular septum thickness (diastolic), *LVDd* left ventricular dimension (diastolic), *LVEF* left ventricular ejection fraction, *PWd* left ventricular posterior wall thickness (diastolic)

Cardiac catheterization revealed that none of the patients had >25 % coronary stenosis, and there were coronary vasomotor abnormalities in 33 patients (54.0 %; epicardial coronary artery spasm, *n* = 13; coronary microvascular spasm, *n* = 4; coronary microvascular dysfunction, *n* = 16). Two of the 13 patients who had epicardial coronary artery spasm (15.3 %) had trivial atherosclerosis (<25 %). A greater proportion of patients were diagnosed as having non-ischemic HF in the LGE-negative group [LGE-negative 17/27 (62.9 %) vs. LGE-positive 11/34 (32.3 %); *p* = 0.017] (Fig. 1d).

**Fig. 2:**
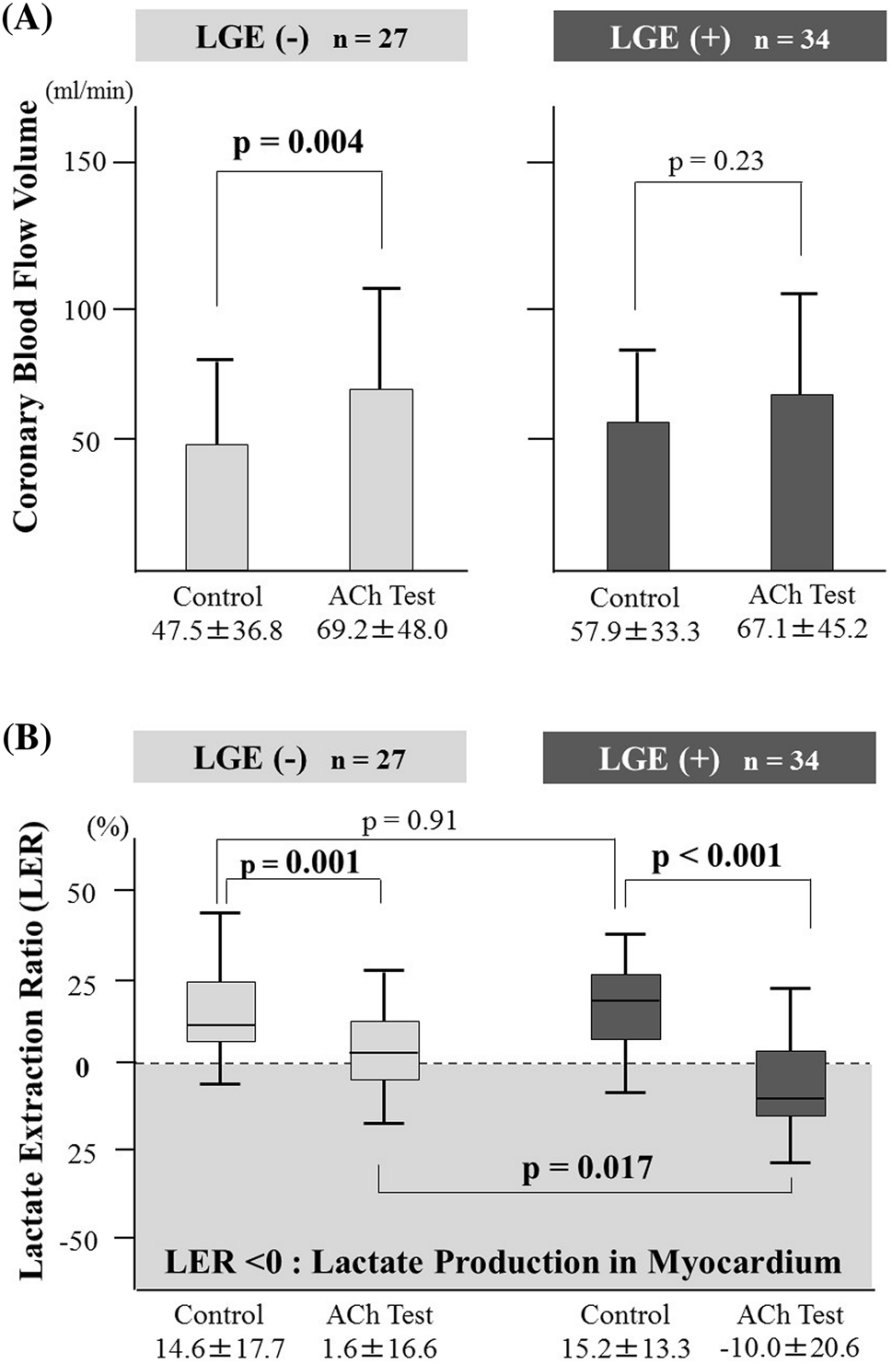
Results of cardiac catheterization. **a** Comparison of the LER in HF with or without LGE. The LER decreased significantly in the LGE-positive during the ACh provocation test. LER of <0 % indicates lactate production by the myocardium. *ACh* acetylcholine, *LER* lactate extraction ratio, *LGE* late gadolinium enhancement. **b** Comparison of the coronary blood flow volume in HF with or without LGE. The coronary blood flow volume increased significantly only in the LGE-negative group during the ACh provocation test

We evaluated the transcardiac LER before and after the ACh test. The LER in the ACh test significantly decreased in both groups (LGE-negative: before vs. after ACh, 14.6 ± 17.7 % vs. 1.6 ± 16.6 %, *p* = 0.001; LGE-positive: before vs. after ACh, 15.2 ± 13.3 % vs. −10.0 ± 20.6 %, *p* < 0.001) (Fig. 2a). Additionally, lactate production significantly increased after ACh provocation only in the LGE-positive group (*p* = 0.017) (Table 4). Thus, transcardiac lactate production occurred during the ACh test only in the LGE-positive group.

**Table 4:** Results of ACh provocation test

	LGE(−) *n* = 27	LGE(+) *n* = 34	*p* value
ECG change	10 (37.0)	13 (38.2)	0.92
Chest pain	4 (14.8)	8 (23.5)	0.40
Lactate production	10 (37.0)	*23 (67.6)*	*0.017*
Epicardial spasm	3 (11.1)	10 (29.4)	0.27

Values are presented as *n* (%)

ECG change, chest pain and epicardial spasm had no difference between two groups

Lactate Production equates to a lactate extraction ratio <0 %

*ACh* acetylcholine, *ECG* electrocardiogram, *LGE* late gadolinium enhancement

The CBFV significantly increased after the ACh provocation test only in the LGE-negative group (before vs. after ACh, 47.5 ± 36.8 vs. 69.2 ± 48.0 ml/min; *p* = 0.004) (Fig. 2b). Nonetheless, there was no significant difference in CFR between the groups (LGE-negative 2.45 ± 1.46 vs. LGE-positive 2.48 ± 1.36; *p* = 0.93).

Univariate and multivariate logistic regression analyses were performed to identify the independent parameters associated with LGE on CMR. Late gadolinium enhancement was significantly correlated with an LER < 0 % after the ACh provocation test (*p* = 0.031; odds ratio, 3.37; 95 % confidence interval 1.12–10.17) (Table 5).

**Table 5:** Univariate and multivariate logistic regression analysis of lge-positive findings in all study patients (*n* = 61)

Varieties	Univariate analysis		Multivariate analysis	
	*p* value	OR (95 % CI)	*p* value	OR (95 % CI)
Age (per year)	0.56	1.39 (0.46–4.19)		
Male (yes)	0.90	1.00 (0.97–1.04)		
Body mass index (per kg/m^2^)	0.38	1.07 (0.92–1.24)		
Hypertension (yes)	0.49	1.57 (0.44–5.64)		
Dilated cardiomyopathy (yes)	*0.049*	*0.32 (0.10*–*1.00)*	0.051	0.31 (0.09–1.17)
Atrial fibrillation (yes)	0.09	0.38 (0.12–1.17)	0.23	0.45 (0.13–1.63)
LVEF at echocardiogram (per %)	0.71	1.01 (0.97–1.05)		
*E*/*e′* at echocardiogram >12 (yes)	0.55	1.36 (0.49–3.81)		
Cardiac index (per ml/min)	0.62	1.26 (0.51–3.11)		
PCWP (per mmHg)	0.51	1.05 (0.91–1.21)		
LER after ACh test ≤0 (yes)	0.019	3.56 (1.23–10.27)	0.031	3.37 (1.12–10.17)
Epicardial spasm (yes)	0.16	2.50 (0.68–9.13)	0.69	1.36 (0.29–6.39)

Lactate production from myocardium (LER < 0) after the ACh test was independently and significantly correlated with LGE positivity

*ACh* acetylcholine, *CI* confidence interval, *LER* lactate extraction ratio, *LGE* late gadolinium enhancement, *OR* odds ratio, *PCWP* pulmonary capillary wedged pressure

Finally, in LGE-positive patients, univariate and multivariate logistic regression analyses showed that a  %LGE of >11.0 % (that is in excess of the median  %LGE) was a significant and independent predictor of a post-ACh LER of <0 % (*p* = 0.042; odds ratio, 6.02; 95 % confidence interval 1.07–33.86) (Table 6).

**Table 6:** Univariate and multivariate logistic regression analyses of an LER of <0 % after the ACh-provocation test in LGE-positive patients (*n* = 34)

Varieties	Univariate analysis		Multivariate analysis	
	*p* value	OR (95 % CI)	*p* value	OR (95 % CI)
Age (per year)	0.30	0.98 (0.93–1.02)		
Male (yes)	0.45	0.51 (0.09–2.99)		
Body mass index (per kg/m^2^)	0.11	1.21 (0.96–1.52)	0.14	1.22 (0.94–1.59)
Hypertension (yes)	0.73	1.30 (0.30–5.54)		
Dilated cardiomyopathy (yes)	0.55	1.56 (0.37–6.62)		
Atrial fibrillation (yes)	0.51	0.56 (0.10–3.10)		
LVEF at echocardiogram (per %)	0.58	1.02 (0.96–1.08)		
*E*/*e′* at echocardiogram > 12 (yes)	0.71	0.76 (0.18–3.23)		
Cardiac index (per ml/min)	0.55	0.67 (0.17–2.55)		
PCWP (per mmHg)	0.28	1.13 (0.91–1.39)		
%LGE ≥ median^*^ (yes)	0.046	5.00 (1.03–24.28)	0.042	6.02 (1.07–33.86)
Epicardial spasm (yes)	0.007	18.47 (2.22–153.44)	0.06	9.26 (0.88–97.83)

%LGE was independently and significantly correlated with an LER < 0 % after the ACh-provocation test in LGE-positive patients (*n* = 34)

%LGE is expressed as the percentage of LGE volume to total left ventricular volume

*ACh* acetylcholine, *CI* confidence interval, *LER* lactate extraction ratio, *LGE* late gadolinium enhancement, *OR* odds ratio, *PCWP* pulmonary capillary wedged pressure

* >11.0 % (=median of %LGE)

## Discussion

This is the first study to have investigated the relationship between microvascular dysfunction assessed in the ACh test and the presence of myocardial fibrosis assessed by CMR in patients with nonischemic HF.

Our major findings were: (a) the patient with HF but without coronary stenosis had coronary vasomotor dysfunction (Fig. 1d); (b) the LER after the ACh provocation test was significantly lower in the LGE-positive group than the LGE-negative group (Fig. 2a); (c) myocardial lactate production (LER < 0 %) after the ACh test, but not epicardial spasm, was independently and significantly correlated with LGE-positivity on CMR (Table 5); and (d) a  %LGE value greater than the median independently and significantly correlated with myocardial lactate production after the ACh test in LGE-positive patients (Table 6).

### Wall tension and LGE in HF patients

Lactate production occurred in myocardial tissue with LGE on CMR in HF without coronary stenosis, and microvascular dysfunction is therefore a potential cause of cardiac ischemia. In the LGE-positive group, however, lactate production increased without a decrease in blood flow during the ACh test.

We and others have also previously examined the relationship between LGE and the transcardiac release of troponin T, and the relationship between LGE volume with LVEDP and transcardiac secretion of B-type natriuretic peptide (BNP) in patients with non-ischemic HF [36, 37]. Pulmonary capillary wedge pressure (PCWP), LVEDP and BNP reflect the extent of cardiac wall tension. Our previous findings appeared to indicate that myocardial fibrosis provokes ongoing myocardial damage, and that the extent of myocardial fibrosis is associated with wall tension in HF [38]. Furthermore, several groups have reported that cardiac wall tension is associated with coronary artery spasm [39, 40]. It has been reported that wall tension and transcardiac troponin T concentration rise during the ACh provocation test [41]. Based on our previous studies and the findings of other investigators, transcardiac troponin T release would likely increase after the ACh provocation test in LGE-positive patients, but we measured only lactate concentration in blood sampled simultaneously from the Ao and the CS in this study. We speculate that wall tension induced focal microvascular spasm in LGE-positive patients. Drugs that act to lower wall tension by modulating LVEDP, PCWP and BNP concentration, such as ACE inhibitors, ARBs, beta blockers and mineralocorticoid blockers, have the potential to ameliorate microvascular dysfunction and cardiac fibrosis in HF, and improve patient outcomes.

### Endothelium-dependent vasomotor and LGE in HF patients

Intra-coronary ACh provocation test is the standard method for diagnosing epicardial coronary artery spasm in patients without coronary artery stenosis. The ACh test is an endothelium-dependent coronary reactivity test used to assess endothelial function and coronary artery spasm. We previously found that patients with coronary microvascular spasm exhibit vascular endothelial dysfunction [22].

In this study, some patients exhibited lactate production after the ACh test without a concomitant reduction in CBFV. We propose that this may be a consequence of a blood flow imbalance that developed due to endothelium-dependent microvascular dysfunction at the LGE site in the coronary artery during the test. Indeed, 64.7 % of the patients in the LGE-positive group exhibited LGE in the LAD area (Fig. 1c). Moreover, logistic regression analyses showed a strong relationship between  %LGE and lactate production in the ACh test (Tables 5, 6).

Based on these study results, we speculate that myocardial fibrosis could indicate the presence of cardiac ischemia, which in turn induces further myocardial fibrosis. Therefore, a vicious circle involving cardiac fibrosis and ischemia may be occurring in HF.

It has been reported that patients with HF have more extensive coronary microvascular rarefaction and myocardial fibrosis than normal patients, and that the progression of coronary microvascular rarefaction correlates with myocardial fibrosis [42]. Therefore, coronary rarefaction may have been occurring in parallel with microvascular dysfunction in LGE-positive patients in this study. In addition, it is reported that pharmacologic treatment with an ACE inhibitors, ARBs and MR blockers reduce cardiac fibrosis and repair microvascular dysfunction in non-ischemic HF [43–46]. Based on these reports, it is essential to achieve optimal medical therapy with an ACE inhibitor (or ARB) and a beta blocker, especially for patients with HF and myocardial fibrosis.

### Study limitations

This study had some limitations. First, the etiology of HF in the cohort was heterogeneous, and the mechanisms of lactate production may have differed among cardiomyopathies. In particular, DCM or other cardiomyopathy differ pathophysiological mechanisms far from HCM. In addition, HCM rarely occur HF and are used different medication from other cardiomyopathy. To establish the extent of this possible limitation, we excluded HCM patients from this study and examined the relationship between LGE and microvascular dysfunction using the same methods. In the patients without HCM (*n* = 50) in this study, The CBFV significantly increased after the ACh provocation test in the LGE-negative group (before vs. after ACh, 47.8 ± 38.1 vs. 72.1 ± 48.7 ml/min; *p* = 0.003, *n* = 26), but the CBFV did not increased after the ACh provocation test in the LGE-positive group (before vs. after ACh, 57.7 ± 35.0 vs. 67.5 ± 44.0 ml/min; *p* = 0.30, *n* = 24). The LER in the ACh test significantly decreased in both groups (LGE-negative: before vs. after ACh, 13.7 ± 18.0 vs. 1.2 ± 16.6 %, *p* = 0.013; LGE-positive: before vs. after ACh, 16.5 ± 14.9 % vs. −11.2 ± 21.6 %, *p* < 0.001), and lactate production significantly increased after ACh provocation only in the LGE-positive group (*p* = 0.021). LGE was significantly and independently correlated with an LER < 0 % after the ACh provocation test in the multivariate logistic regression analyses (*p* = 0.009; odds ratio, 13.47; 95 % confidence interval 1.90–95.24). These results in the patients without HCM (*n* = 50) were almost same as the results of all study patient (*n* = 61) (Fig. 2a, b; Table 5). Our results of HF patients with (*n* = 61) or without HCM (*n* = 50) indicated that LGE on CMR correlated with lactate production after the ACh provocation test and coronary microvasomotor abnormality.

Second, we enrolled a relatively small number of patients from a single center, limited to patients with HF but without coronary stenosis. Many patients declined to undergo cardiac catheterization, as they had no chest pain; consequently we were only able to recruit 61 patients in 7 years. Indeed, 35 patients rejected invasive cardiac catheterization because they were diagnosed as having non-ischemic heart disease before hospitalization.

Third, 21 patients (34.4 %) could not tolerate ACE inhibitor, ARB or beta blocker therapy even at low doses because of hypotension. It has been reported that ACE inhibitors, ARBs and beta blockers influence microvascular function [47]; however, both would be expected to have less of an impact on microvascular function than calcium channel blockers or nitrates.

Fourth, we examined coronary microvascular function in the LAD alone, as in our opinion it would have been too time-consuming and invasive to perform an ACh provocation test in both coronary arteries. Indeed, 22 of the 34 LGE-positive patients (64.7 %) exhibited LGE in the LAD area (Fig. 1c).

Fifth, the quantitative analysis of myocardial fibrosis by LGE is that the fibrotic process is often diffuse in nonischemic cardiomyopathies, and the extent of LGE may be underestimated in such patients because of the lack of normal, non-fibrotic myocardium as a reference.

Finally, we used LGE to evaluate myocardial fibrosis rather than extracellular volume fraction (ECV). Although ECV is now an extensively validated method of measuring myocardial fibrosis, this was not the case at the start of our study [48–54]. To minimize the influence of the limitations of measuring LGE, we undertook CMR only when the patients’ symptoms and hemodynamic statuses were stable.

## Conclusion

Late gadolinium enhancement on cardiac magnetic resonance is correlated with lactate production in the acetylcholine provocation test. The presence of late gadolinium enhancement indicates ischemic damage due to coronary vasomotor abnormality. Late gadolinium enhancement in patients with HF may indicate worsening of HF. Further research is required to understand the role of myocardial fibrosis in HF more fully, and to identify new means of further improving patient outcomes.

Translational Outlook: Patients with nonischemic HF exhibiting LGE on CMR likely have ischemia caused by vasomotor abnormality.
